# Simulating domain architecture evolution

**DOI:** 10.1093/bioinformatics/btac242

**Published:** 2022-06-27

**Authors:** Xiaoyue Cui, Yifan Xue, Collin McCormack, Alejandro Garces, Thomas W Rachman, Yang Yi, Maureen Stolzer, Dannie Durand

**Affiliations:** Computational Biology, Carnegie Mellon University, Pittsburgh, PA 15213, USA; Computational Biology, Carnegie Mellon University, Pittsburgh, PA 15213, USA; Department of Biological Sciences, Carnegie Mellon University, Pittsburgh, PA 15213, USA; Computational Biology, Carnegie Mellon University, Pittsburgh, PA 15213, USA; Department of Biological Sciences, Carnegie Mellon University, Pittsburgh, PA 15213, USA; Department of Biological Sciences, Carnegie Mellon University, Pittsburgh, PA 15213, USA; Computational Biology, Carnegie Mellon University, Pittsburgh, PA 15213, USA; Computational Biology, Carnegie Mellon University, Pittsburgh, PA 15213, USA; Department of Biological Sciences, Carnegie Mellon University, Pittsburgh, PA 15213, USA; Department of Biological Sciences, Carnegie Mellon University, Pittsburgh, PA 15213, USA; Department of Biological Sciences, Carnegie Mellon University, Pittsburgh, PA 15213, USA

## Abstract

**Motivation:**

Simulation is an essential technique for generating biomolecular data with a ‘known’ history for use in validating phylogenetic inference and other evolutionary methods. On longer time scales, simulation supports investigations of equilibrium behavior and provides a formal framework for testing competing evolutionary hypotheses. Twenty years of molecular evolution research have produced a rich repertoire of simulation methods. However, current models do not capture the stringent constraints acting on the domain insertions, duplications, and deletions by which multidomain architectures evolve. Although these processes have the potential to generate any combination of domains, only a tiny fraction of possible domain combinations are observed in nature. Modeling these stringent constraints on domain order and co-occurrence is a fundamental challenge in domain architecture simulation that does not arise with sequence and gene family simulation.

**Results:**

Here, we introduce a stochastic model of domain architecture evolution to simulate evolutionary trajectories that reflect the constraints on domain order and co-occurrence observed in nature. This framework is implemented in a novel domain architecture simulator, DomArchov, using the Metropolis–Hastings algorithm with data-driven transition probabilities. The use of a data-driven event module enables quick and easy redeployment of the simulator for use in different taxonomic and protein function contexts. Using empirical evaluation with metazoan datasets, we demonstrate that domain architectures simulated by DomArchov recapitulate properties of genuine domain architectures that reflect the constraints on domain order and adjacency seen in nature. This work expands the realm of evolutionary processes that are amenable to simulation.

**Availability and implementation:**

DomArchov is written in Python 3 and is available at http://www.cs.cmu.edu/~durand/DomArchov. The data underlying this article are available via the same link.

**Supplementary information:**

Supplementary data are available at *Bioinformatics* online.

## 1 Introduction

Simulation is an essential technique for studying protein evolution, where most processes of interest act on the time scales that exceed a human lifetime. However, modeling the evolution of multidomain proteins poses special challenges.

**Fig. 1.**
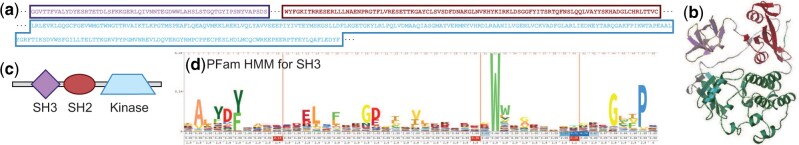
Example multidomain protein: proto-oncogene tyrosine-protein kinase Src in human. (**a**) Domains in the sequence are identified by PFAM (Mistry *et al.*, 2021) HMMs: SH3 (PF00018), SH2 (PF00017), and a protein tyrosine kinase (PF07714). Sequence in linker regions represented as (⋯). (**b**) The 3D structure of Src, with the SH2, SH3, and kinase folds shown in purple, red, and blue. (**c**) Src domain architecture, showing its constituent domains in N- to C-terminal order. (**d**) A sequence LOGO for the PFAM SH3 domain model.

Multidomain proteins are mosaics of sequence segments that encode structural or functional modules (Fig. 1), called *domains*. Domains act as independent units that may be found in many, otherwise unrelated proteins. Modular domains are able to fold correctly in multiple sequence contexts.

The domain content of a protein sequence (Fig. 1a) can be determined by scanning a database of probabilistic domain models that encode the variation in the residue observed at each position of a multiple alignment of a domain superfamily (Blum *et al.*, 2021; Letunic *et al.*, 2006; Lu *et al.*, 2020; Mistry *et al.*, 2021). The result of such a scan is (Fig. 1c) the sequence of ‘tokens’ (e.g. domain names or ids) representing constituent domains in N- to C-terminal order (Vogel *et al.*, 2004). In this *domain architecture* representation, the amino acid sequence of the domain instances is ignored, as is the sequence of the linker regions separating the domains.

### 1.1 Multidomain evolution

**Fig. 2.**
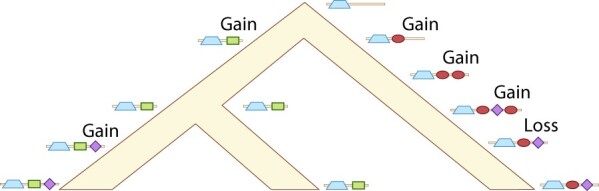
Schematic showing changes in domain architecture via insertion, duplication, and deletion of domains

The domain content of a gene evolves via domain gain and loss (reviewed in Han *et al.*, 2007; Moore *et al.*, 2008; Vogel *et al.*, 2004), giving rise to families that encode related proteins with variable domain content (Fig. 2). The modular nature of multidomain sequences imposes selective forces on the evolutionary process; i.e. the function and structure of individual domains, the interactions between domains, and the overall integrity of the protein must all be preserved.

These constraints are clearly apparent in domain and protein sequences observed in nature. Multiple alignments of sequences encoding a particular domain superfamily exhibit a characteristic pattern of conserved sites (Fig. 1d), consistent with its function and interaction requirements. Constraints acting on domain order and composition are also readily apparent; only a tiny fraction of possible domain combinations is observed in nature (Apic *et al.*, 2003; Bashton and Chothia, 2002; Kummerfeld and Teichmann, 2009; Weiner III *et al.*, 2006). For example, the two most common SUPERFAMILY domains in primate genomes, the Zinc Finger (57667) and the Immunoglobulin (48726) domains, never co-occur in the same protein.

### 1.2 Simulating protein family evolution

Single domain protein family evolution entails changes on two levels of organization: gene events and sequence evolution. Birth–death models, originally designed to model changes in population size (Kendall, 1949), have been adapted to modeling the expansion and contraction of a gene family via gene events (duplications, transfers, and losses). Simulation of these events on a species tree results in a gene tree, wherein each divergence corresponds to a specific gene event or co-divergence with speciation. Gene family sequences can then be produced by generating an ancestral sequence at the root of the gene tree and simulating substitutions along its branches. The result is a set of ‘present-day’ sequences with a known evolutionary history that can be used to test the accuracy of alignment and phylogeny reconstruction software. Some simulators, such as GenPhyloData (Sjöstrand *et al.*, 2013), SimPhy(Mallo *et al.*, 2016), and Zombi (Davín *et al.*, 2020), support simulation of both processes by combining a birth-death model of changes in family content and a stochastic model of sequence evolution in a unified framework.

Multidomain evolution requires modelling changes on three levels of biological organization (gene events, domain events, and sequence evolution).

1. Juxtaposing gene and domain events on a species tree provides a description of the temporal relationships between events on multiple levels of biological organization. As before, gene trees are generated by simulating gene events on a species tree. Simulating domain events (duplications, insertions, and losses) on a gene tree gives rise to genes represented as mosaics of domain segments, as well as domain trees, one for each domain family encoded in the gene family.
2. This results in mosaics of segments that have different evolutionary histories. On the sequence evolution level, these segments correspond to sequences that are evolving at different rates.

This model can be made more realistic by modeling each domain-encoding segment as an instance of a genuine domain family. This imposes two additional requirements:

3. Functional and structural constraints acting on the evolution of each domain sequence preserve its characteristic sequence motifs, e.g. the propensity for alanine at Position 2, aromatic amino acids at Position 6, and tryptophan at Position 32 in SH3 (Fig. 1d).
4. Changes in domain architecture resulting from domain gains or losses must reflect the constraints on domain order and co-occurrence that are observed in nature, such as the absence of genes encoding both a Zinc Finger domain and an Immunoglobulin domain in the same protein.

A comprehensive multidomain protein simulator that supports all four goals has yet to be developed. Several systems have been developed that realize one or more of these goals. The simulator indel-Seq-Gen (Strope *et al.*, 2009) allows for different evolutionary constraints in different regions of a sequence (Goal 2). SaGePhy (Kundu and Bansal, 2019) simulates both gene and domain events by applying the GenPhyloData simulator (Sjöstrand *et al.*, 2013) at two levels of organization, first to simulate gene trees on a species tree and then to simulate domain trees on each gene tree. SaGePhy links phylogenetic histories between the species, gene, and domain trees (Goal 1) and generates mosaics of sequence segments (Goal 2). However, the individual segments are not treated as representations of genuine domains (e.g. from PFAM) with sequence characteristics resulting from evolution under structural or functional constraints.

REvolver (Koestler *et al.*, 2012) models the evolution of a mosaic of sequence segments that correspond to genuine domain families, interspersed with linker sequences. Its sequence evolution model imposes the domain-specific constraints encoded in the HMM of each domain superfamily and maintains domain-specific length distributions. REvolver preserves the characteristic features of each domain (Goal 3) and supports a mosaic organization (Goal 2), but does not model an ongoing process of domain gain and loss.

None of these models captures the constraints acting on domain order and adjacency (Goal 4). The problem of modeling the constraints on domain order and adjacency during domain architecture evolution does not arise in SagePhy, because sequence segments in SagePhy do not correspond to genuine domains. Sequence segments in REvolver do correspond to genuine domains, but REvolver does not model explicit domain events that modify domain architectures.

### 1.3 Our contributions

Here, we present a domain architecture simulator specifically designed to generate domain architectures that mimic the constraints on domain order and co-occurrence observed in nature (Goal 4). DomArchov is a Python implementation of this design. To our knowledge, no algorithms or software that address this problem are currently in existence.

**Fig. 3.**
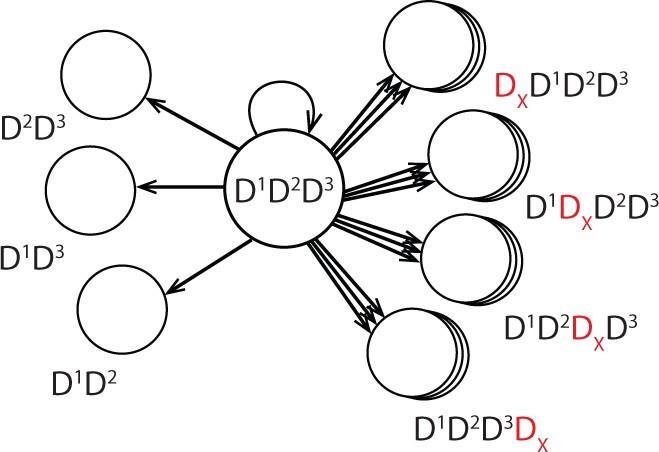
The state transition diagram showing states adjacent to a DA of length *n *=* *3. Each stack of circles on the right represents the *N_D_* states that can be reached by a domain gain at the associated position.

In our model, each state corresponds to a domain architecture (DA), i.e., to an ordered list of domains in N- to C-terminal order. Two states are adjacent if they differ by a single domain (Fig. 3). State transitions, therefore, correspond to the gain or loss of a specific domain at a specific location. We require a stochastic procedure that, with high probability, visits states that correspond to domain architectures with properties that are typical of genuine domain architectures.

The birth-death formalism, widely used to simulate gene events, is not sufficiently expressive to capture constraints acting on domain order and adjacency. States in the birth-death model correspond to family size. Each state is adjacent to two neighboring states corresponding to a birth event, which increments family size, and a death event, which decrements family size. State transition probabilities and the distribution of waiting times between events are fully determined by the birth and death rates, which are explicit state-independent parameters, and a single state parameter, the family size. Thus, birth-death is a single-dimensional model and benefits from various properties that facilitate efficient simulation. In particular, the history of birth and death events can be simulated by repeatedly sampling from the waiting time distribution using the Gillespie algorithm.

In contrast, in DomArchov, states of the system are defined by a sequence of domains. The probability of gaining or losing a domain depends on the specific location of the proposed change in the current DA. For this model, a different simulation strategy is required to account for high dimensionality and transition probabilities cannot be expressed as a simple function of state-independent event rates. To address this problem, we developed a novel stochastic model, described in detail in Section 2, that simulates the evolutionary trajectory of a DA using the Metropolis–Hastings algorithm.

DomArchov can be applied at multiple time scales. In the stationary regime, DomArchov can be used to sample DAs according to an (unknown) equilibrium distribution to investigate evolutionary processes, test competing hypotheses or probe steady-state behavior. On much shorter, non-equilibrium timescales, DomArchov can be applied to generate evolutionary trajectories, producing datasets with ‘known’ histories for testing, validation, and comparison of evolutionary algorithms and software (e.g. Fig. 2).

DomArchov provides an important contribution for studies that use the DA abstraction as the primary data structure. The DA abstraction is widely used to probe questions of protein evolution, including the co-occurrence and variation in domain repertoire across taxonomic lineages (Cromar *et al.*, 2016; Dohmen *et al.*, 2020; Karev *et al.*, 2004; Tordai *et al.*, 2005; Ye and Godzik, 2004), plasticity in domain order (Bashton and Chothia, 2002; Kummerfeld and Teichmann, 2009; Weiner III *et al.*, 2006), domain occurrence graphs (Cromar *et al.*, 2014; Karev *et al.*, 2002; Przytycka *et al.*, 2006; Vogel *et al.*, 2005), and domain promiscuity, i.e. the propensity of a domain to co-occur with many other domains (Basu *et al.*, 2008, 2009; Cohen-Gihon *et al.*, 2011; Cromar *et al.*, 2014; Marcotte *et al.*, 1999). DomArchov provides, for the first time, a simulation engine to complement such studies.

DomArchov is a significant step towards the development of a comprehensive simulator for multidomain protein evolution. By modeling constraints on domain architectures that are not captured by any other software, DomArchov complements a rich body of work for simulating protein evolution on other levels of biological organization. DomArchov can be combined with simulators that model gene tree topologies and amino acid sequence evolution to create hierarchical models of multidomain protein evolution that reflect the multiple scales on which these families evolve. For example, following generation of a gene tree using a birth-death simulation of gene events on a species tree, DomArchov is applied to simulate the evolution of a domain architecture along the branches of that tree. In this scenario, an artificial DA is instantiated at the root of the gene tree. Next, an amino acid sequence is instantiated for each domain based on the HMM model for that domain. Linker sequences inserted between domains are based on the background distributions of sequence composition and linker lengths. DomArchov is then applied to evolve the DAs along each gene tree branch, progressing from the root to the leaves. Finally, domain sequences are evolved using a simulator that constrains evolution based on the domain model. Linker sequences are modeled using a standard sequence evolution simulator.

Transitions between states in Metropolis–Hastings depend on the ratio of state probabilities. Because there is no general theory of ‘modular collaboration’ that would allow us to calculate state probabilities directly from domain architectures, we estimated state probabilities using a first-order approximation wherein the probability of observing a domain depends only on the identity of the domain that immediately precedes it. A key question is whether an algorithm that makes decisions based only on the immediate genetic neighborhood can nevertheless recapitulate longer range domain architecture properties. To investigate this question, we simulated DAs using four training sets derived from four disparate metazoan lineages and, in each case, compared the simulated DAs with genuine architectures taken from the same lineage as the training data.

We examined properties including those that are not explicitly reflected in domain pair statistics used as training data: domain architecture probabilities, the number of copies in tandem arrays of repeated domains, domain promiscuity, and the propensity of domain pairs to co-occur in the same protein. Remarkably, although state transitions are based only on local information about domain order and proximity, DomArchov is able to elicit global properties of domain architectures.

## 2 Materials and methods

### 2.1 Simulator design

In our simulator, the evolution of a domain architecture is modeled as a Markov chain (Algorithm 1). Each state is a DA, where each domain instance is represented by its superfamily ID. Let D be the set of *N_D_* domain superfamilies in the training data and *D_d_* be an instance of domain superfamily d∈D. A DA of length *n* is denoted by $D_{\phi }^{0}D_{d_{1}}^{1}D_{d_{2}}^{2}\cdots D_{d_{n}}^{n}D_{\phi }^{n+1}$, where the superscript indicates the position in the DA and the subscript gives the domain superfamily ID. The NULL domain, $D_{\phi }$, is used to mark the termini of the DA.

Algorithm 1: DA simulator (DomArchov) pseudocode

**Input**: DA_0_, initial DA; *T*, maximum iterations

**1**DA = DA_0_

**2 for** *t* =0  **to** *T* **do**

**3  **n=length(DA)

4 *r* = U[0,1]** **//** **Choose new event uniformly at random

5  **if** *r* $>\frac{1}{N_{D}}$  **then **// Insert

6 **  ***i* =RandomNumber(*0, n + *1)

7 **  ***d* =RandomNumber(*0, N_D_*)

8 **  **DA′  =insert(*DA, d, i*)

9 ** else **// Delete

10 **  ***i* =RandomNumber(*0, n*)

11 **  **DA′=delete(DA*, i*)

12 ** if**  n==0  **then** next;** **// Prevent extinction

13  **else** with probability min(*1*, p(DA′)/p(DA)), DA  =DA′

State transitions correspond to *domain gains* and *domain losses*. Given a DA of length *n*, there are *n* possible deletion sites and *n *+* *1 possible insertion sites. For each insertion site, there are *N_D_* possible insertions, resulting in $n+(n+1)N_{D}$ adjacent states (e.g. Fig. 3). Generally, *N_D_* is on the order of a few thousand, and *n* can reach 100, resulting in the possible number of adjacent states reaching on order of 10^5^. When *n *=* *1, deletions are forbidden to avoid extinction. Thus, architectures composed of a single domain have $2N_{D}$ adjacent states.

At each iteration, a new state is proposed, uniformly at random, from all states that are adjacent to the current state. DomArchov allows the user to modify the ratio of gain to loss state proposals; however, deviation from uniformly distributed state proposals will disrupt the convergence properties of the model. First, an event type (gain or loss) is selected. Since the number of gain states exceeds the number of loss states by a factor of roughly *N_D_*, the probability of selecting a gain event is set to be *N_D_* times greater than a loss event to ensure that all states transitions are considered with equal probability. Once the event type has been determined, a state is selected uniformly at random among the adjacent states corresponding to that event.

Proposed states are accepted or rejected in accordance with the Metropolis–Hastings algorithm. The probability of a transition from state *s* to an adjacent state *t* is:

(1) $$P[s,t]=\min(1,\frac{p_{t}}{p_{s}}),$$

where *p_s_* and *p_t_* are the probabilities of the DAs associated with states *s* and *t*, respectively. The probability of observing a given DA is estimated from domain pair frequencies using a first-order approximation, in which the probability of observing a domain is only dependent on the domain that immediately precedes it. For example, the probability of observing state $s=D_{\phi }^{0}\cdots D_{d_{i-1}}^{i-1}D_{d_{i}}^{i}\cdots D_{\phi }^{n+1}$ is approximated by:

(2) $$p_{s}\approx p(D_{\phi }^{0})p(D_{d_{1}}^{1}|D_{\phi }^{0})\cdots p(D_{d_{i}}^{i}|D_{d_{i-1}}^{i-1})\cdots p(D_{\phi }^{n+1}|D_{d_{n}}^{n}).$$

Let state *t* be the DA that results from the gain of $D_{d_{x}}$ between $D_{d_{i-1}}^{i-1}$ and $D_{d_{i}}^{i}$ in *s*:

$$t=D_{\phi }^{0}\cdots D_{d_{i-1}}^{j-1}D_{d_{x}}^{j}D_{d_{i}}^{j+1}\cdots D_{\phi }^{n+1}.$$

Then, the ratio of the probabilities of *t* and *s* can be approximated by the ratio of two first-order approximations. Most of the terms in the numerator and the denominator of that ratio cancel, yielding:

(3) $$\frac{p_{t}}{p_{s}}\approx \frac{p(D_{d_{x}}|D_{d_{i-1}})p(D_{d_{i}}|D_{d_{x}})}{p(D_{d_{i}}|D_{d_{i-1}})}.$$

Similarly, the transition probability for deletion of the domain at position *i* is:

(4) $$\frac{p_{t}}{p_{s}}\approx \frac{p(D_{d_{i+1}}|D_{d_{i-1}})}{p(D_{d_{i}}|D_{d_{i-1}})p(D_{d_{i+1}}|D_{d_{i}})}.$$

The conditional probability $p(D_{d_{i}}|D_{d_{i-1}})$ is estimated from bigram frequencies in genuine data:

(5) $$p(D_{d_{y}}|D_{d_{x}})=\frac{C(D_{d_{x}}D_{d_{y}})}{\sum_{d_{k}\in D}C(D_{d_{x}}D_{d_{k}})},$$

where $C(D_{d_{x}}D_{d_{y}})$ is the number of observed $D_{d_{x}}D_{d_{y}}$ pairs.

The matrix of domain bigram frequencies is very sparse. A pseudocount is required to account for bigrams that have not been previously observed. This also ensures that the state space is connected and that the Markov chain is therefore irreducible. Based on the Add-k smoothing method (Jurafsky and Martin, 2008), a small value *k* is added to the count of each bigram. To offset the fact that the number of pseudocounts added per domain is $O(N_{D})$, in our analysis, we chose $k=\frac{1}{N_{D}}\approx 0.0009$. This value prevents over-smoothing, while still allowing un-observed domain combinations to occur given reasonably long simulations.

### 2.2 Implementation

DomArchov is written in Python 3 and is composed of three modules:

1. Raw data pre-processing of genomes downloaded from SUPERFAMILY, with domain architectures as the output;
2. Pre-calculation of domain statistics used when calculating transition probabilities; and
3. The simulator.

The pre-processing module extracts domain annotations to calculate the domain architectures as well as the domain alphabet, bigrams, and trigrams, and their associated counts. The pre-calculation module then calculates information that will be used repeatedly during simulations, such as the domains that are observed between domain pairs, the number of domains observed following a given domain, domains that are never observed in a multidomain context, etc. These two modules are used to set up the suitable environment for the simulator and are the basis for calculating transitions. They only need to be run once for a given set of genomes.

The simulation module takes as input a JSON file that includes the user-defined parameters for the simulation, including the starting DA, chain length, number of replicates, and pseudocount *k*. A batch of simulations can also be run over a defined range for a given variable. Output includes the final DA simulated for each replicate; these data can be used to calculate the same statistics as the input genomes. Summary statistics over all replicates are also written to file, and options exist to output the evolutionary trajectory (states in the Markov chain) for each replicate.

## 3 Results

Our goal is to build a domain architecture simulator that can generate artificial DAs with properties that are comparable to those of genuine DAs. We evaluated DomArchov empirically using four training datasets derived from four metazoan lineages: 7 primate genomes, 11 fish genomes, 13 Drosophila genomes, and 4 cnidarian genomes (see Supplementary Table S1). We focused on metazoan datasets because of the size and complexity of metazoan multidomain protein families (Ye and Godzik, 2004).

For each of the four datasets, we carried out simulation experiments for chain lengths ranging from T=7.5K to T=12.8M, where *T* is the number of states proposed by the Metropolis–Hastings algorithm (see Algorithm 1). Each experiment consisted of a suite of replicates equal in number to the number of unique DAs in the corresponding training set (Supplementary Material S1). In this study, each replicate was initiated with a randomly selected state (a single domain). The final DA in each replicate, following *T* attempted state transitions, was tabulated, generating a set of DAs for evaluation. Statistics of proposed and accepted domain additions and deletions were also recorded. For convergence tests, we kept detailed traces for 1000 replicates from primate DA simulations with T=3.2M, including DA length and event positions in the DA.

### 3.1 Training data

Domain architectures were downloaded from the online SUPERFAMILY database, version 1.75 (Gough and Chothia, 2002). Each genomic annotation file includes the species identifier and, for each domain, the sequence in which it is found, the first and last amino acid positions for the domain model, and the domain family and superfamily identifiers. SUPERFAMILY annotations were chosen because the domain models are based on the SCOP structural classification (Andreeva *et al.*, 2020) and related domain instances are grouped in a hierarchical organization, where families that share a common ancestor are grouped into a superfamily.

Domain bigram statistics were obtained by first extracting a list of all DAs from the genome annotation file. To avoid over-representation of bigrams caused by gene duplication, rather than domain-level changes, the set of unique DAs was then determined from this list. Observed monogram and bigram frequencies, as well as first-order conditional probabilities for bigrams, were then calculated from this set of unique DAs. Training data summary statistics are provided in Table 1.

**Table 1.** Summary statistics for four training datasets

	Primates	Fish	Fly	Cnidaria
Unique DAs	7144	8985	5483	6271
Domains	1132	1131	1017	1159
Unique bigrams	2865	3357	2884	3533
Mean DA length	5.4	6.2	5.2	3.6
Median DA length	3	4	3	3

### 3.2 Convergence

**Fig. 4.**
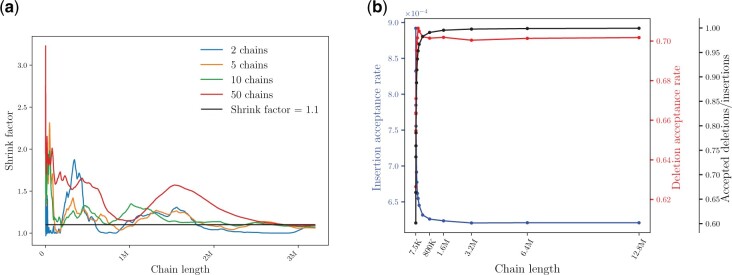
MCMC convergence assessment. (a) Gelman Rubin diagnostic applied to DA lengths sampled every 100 iterations with the primate dataset. (b) Event acceptance rate.

We first considered the convergence properties of the simulator and the stability of the sampling process. A formal assessment of model convergence was obtained using the Gelman and Rubin (1992) diagnostic for multiple parallel chains, which compares the between-chain variance and within-chain variance and reports the ‘shrink’ factor, a measure of the agreement between these variances. For convergence tests, we focused on the primate dataset, which contains the largest and most complex domain architectures. An overall shrink factor below 1.1 is typically considered evidence of approximate convergence (Brooks *et al.*, 2011). In our tests with primate data (Fig. 4a), we obtained final shrink factors below 1.1 for all comparisons when T=3.2M. When 50 chains were compared, the shrink factor dropped below 1.13 with Drosophila data and to 1.16 with fish. For all other cases, we obtained final shrink factors below 1.1.

To assess the stability of the simulator, we examined the event acceptance rate; that is the fraction of proposed state transitions that are accepted by the Metropolis–Hastings algorithm (Fig. 4b). The frequencies of both accepted gain and accepted loss events rapidly reached a steady state. Proposed deletions are accepted much more readily than proposed gains, which is consistent with the theoretical transition probabilities (Equations 3 and 4). However, gain proposals exceed deletion proposals by three orders of magnitude (Algorithm 1), which offsets this discrepancy. Moreover, the ratio of accepted gains to accepted losses rapidly approaches one (Fig. 4b).

### 3.3 Recapitulation of genuine domain architecture properties

#### 3.3.1 Domain architecture lengths

**Fig. 5.**
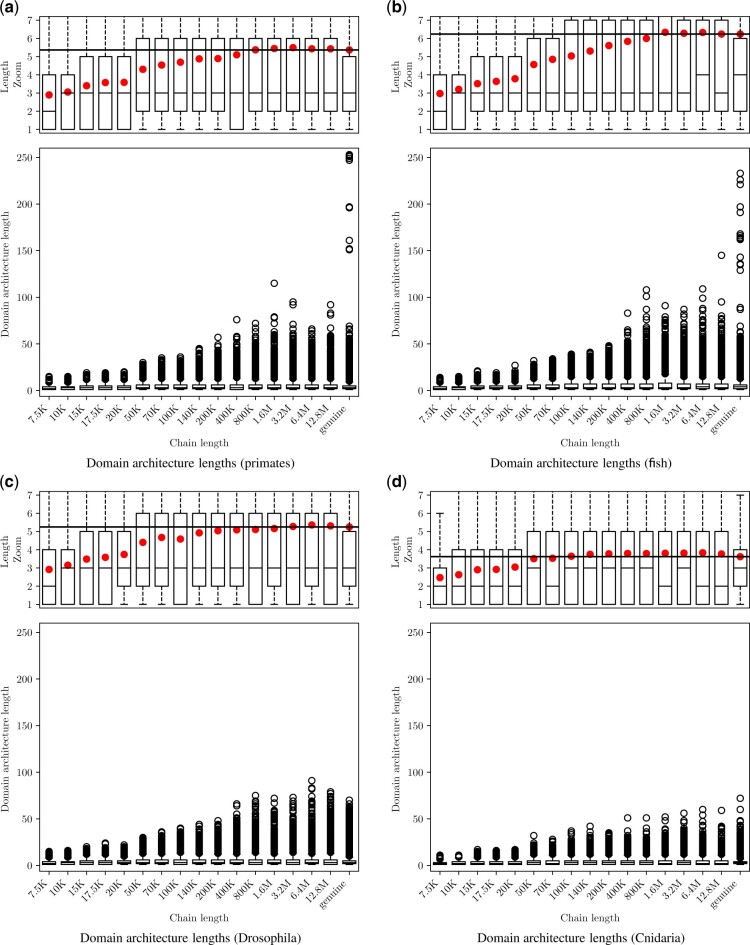
Final DA length as a function of chain length (horizontal axis not to scale). Top panels: Close-up view of the same distribution. Mean DA length shown as solid dots. Horizontal line represents mean length of genuine DAs. Length distributions of genuine DAs are plotted in the rightmost columns. (a) Primates. (b) Fish. (c) Drosophila. (d) Cnidaria.

We first considered the distributions of simulated DA lengths as a function of MCMC chain length, *T*, where *T* varies from 7.5 K to 12.8 M proposed state changes. We observe the same trends in all four datasets (Fig. 5). The simulated and genuine DA length distributions agree well: In all cases, the mean simulated DA length continues to grow until it reaches a plateau close to the observed mean (black horizontal line.) At this point, the numbers of deletions and insertions are roughly equal so that the average length of simulated DAs does not grow indefinitely (Fig. 4b). Median simulated DA lengths also closely approximate those in genuine data for sufficiently large *T*.

Interestingly, in the two vertebrate lineages, the longest domain architectures in genuine data are much more extreme than those in the simulated data. For example, the longest DA observed in any replicate in the primate simulation contained 115 domains. There are 10 primate DAs that exceed this length, with the longest, Titin, containing 253 domains. This is also true for fish, where 10 genuine DAs are longer than the longest simulated DA. For the two invertebrate datasets, we did not observe more extreme outliers.

The simulated DA length statistics demonstrate the stability of our model. The Markov model achieves a balance between domain gain and domain loss that produces realistic DA lengths.

#### 3.3.2 Domain architecture probabilities

The probabilities of the genuine and simulated DAs were also calculated. These probabilities were estimated using the first-order approximation given in Equation (2). As expected, the probability of a DA is inversely related to its length (Supplementary Fig. S1). Comparing the DA lengths and probabilities reveals that DAs in simulated data largely recapitulate genuine DA lengths and probabilities. As seen before, the exception lies with the longer DAs observed in the genuine primate and fish genomes.

To mitigate the effect of these extreme DA lengths, the longest 1% of DAs were removed from the simulated and genuine data, independently. The distributions were then compared with Q-Q plots (Supplementary Fig. S2) and quantile correlation (see the DA probability quantile correlation coefficients in Table 2). The distributions are highly correlated and appear similar, especially for DAs with probabilities in the tails of the distribution; however, the middle quantiles of genuine DAs skew to the right, with the median a higher probability in genuine data compared with simulated.

**Table 2.** Domain combination statistics in genuine and simulated DAs (T=3.2M) are highly correlated (Pearson correlation coefficient, p<1e−15 for all tests)

	Primate	Fish	Drosophila	Cnidaria
Singleton frequency	0.998	0.999	0.994	0.994
Bigram frequency	0.997	0.998	0.994	0.988
Trigram frequency	0.969	0.968	0.986	0.962
Pair co-occurrence	0.868	0.832	0.944	0.888
Unique neighbors	0.925	0.922	0.918	0.946
Wtd bigram promiscuity	0.843	0.870	0.925	0.821
Mean tandem array length	0.889	0.927	0.935	0.942
DA probability quantiles	0.976	0.973	0.964	0.984

The differences in distributions may be due to the fact that the simulator samples shorter DAs with lower probability more uniformly than is observed in genuine data (Supplementary Fig. S1). This may be due to model choices, such as first-order approximation, or factors acting on real proteins that are not represented in the simulator, such as selection.

#### 3.3.3 Frequencies of domains and domain combinations

We next investigated how well the model recapitulates constraints acting on domain order and adjacency. If the simulation procedure, with a first-order approximation as the modeling choice, is successful in mimicking these constraints, then the properties of individual domains and short domain combinations in simulated DAs should be similar to those in genuine genomes. As a sanity check, we verified the agreement between the frequencies of domains, domain bigrams, and domain trigrams in the genuine and simulated DAs (T=3.2M). To probe this question further, we considered the propensity of a pair of domains to co-occur at any location in the same protein. Indeed, these quantities are in very good agreement in all four datasets (Table 2), suggesting that the simulation procedure does preserve those constraints.

We further investigated domain ‘promiscuity’, the tendency of some domains to be found in combination with many other domains in different domain architectures (Marcotte *et al.*, 1999). We considered two metrics: the number of unique neighbors of each domain and the weighted bigram promiscuity of the 100 most promiscuous domains, proposed by Basu *et al.* (2008). These quantities also show a strong correlation between the genuine and simulated sets of DAs (Table 2).

#### 3.3.4 Dependence of domain gains and losses on ordinal position

Several studies, using a variety of comparative approaches, report evidence that evolutionary events occur more commonly at the termini of multidomain proteins than in the interior (Björklund *et al.*, 2005; Buljan and Bateman, 2009; Buljan *et al.*, 2010; Kummerfeld and Teichman, 2005; Snel *et al.*, 2000; Weiner III *et al.*, 2006). Several mutational processes that modify domain architectures result in modifications at the N- or C-terminal of a protein, including gene fusion, acquisition of premature stop codons, and acquisition of an alternative transcriptional or translational start signal. Proteins may also be more resilient to changes at the termini, since those regions are often located on the surface of the folded protein, rather than contributing to the structural integrity of the protein core (Buljan and Bateman, 2009).

**Fig. 6.**
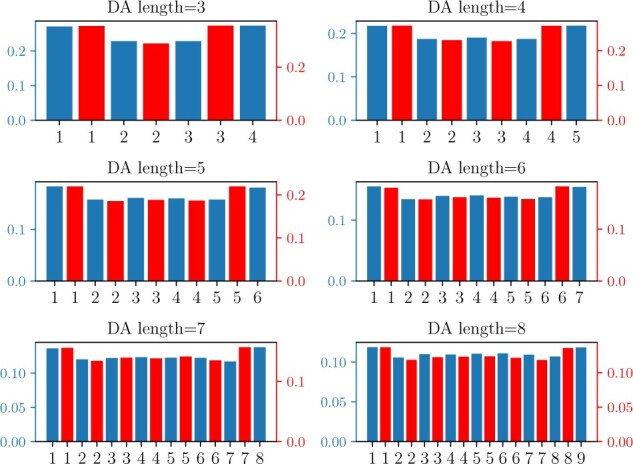
Frequency of accepted gain (left axis) and loss (right axis) positions; bars for gains (blue) and losses (red) are interleaved, starting with gains.

To determine whether the simulator recapitulates this behavior, we tabulated the positions of accepted gain and loss events. Figure 6 shows the frequency of accepted events at each position in simulated DAs of lengths 3–8 observed in 1000 primate replicates (T=3.2M). In all cases, there is a clear preference for terminal over internal gains and losses.

#### 3.3.5 Duplications

Domain repeats are prevalent in multidomain proteins and play important roles in binding specificity and structural integrity (Björklund *et al.*, 2006, 2010; Schüler and Bornberg-Bauer, 2016). In DomArchov, domain duplications are implicitly modeled by gains of multiple instances of the same domain superfamily at adjacent positions. Since acceptance probabilities are driven by observed bigram frequencies, domains with a high propensity for tandem arrangements in the training data are also expected to have an elevated propensity for insertion next to members of the same superfamily in the simulator.

To determine whether this is sufficient to generate DAs that exhibit patterns of tandem repeats similar to those in genuine proteins, we compared tandem array lengths in simulated and genuine domain architectures. Our results show that tandem array lengths are highly correlated (Table 2), although the association is not as strong as the agreement between domain combination frequencies.

We further asked whether domains frequently observed in tandem arrays in genuine data also have a tendency to form tandem arrays in the simulated data. We ranked domain superfamilies by their tandem copy number aggregated over all unique domain architectures. The same 10 domain superfamilies ranked highest in both the genuine and the simulated data (Table 3). This suggests that the DomArchov prototype, which derives domain gain probabilities from bigram frequencies, preserves the propensity to form tandem arrays without explicitly modeling duplication.

**Table 3.** The 10 domains with the most copies in tandem arrays

	Genuine		Simulated	
Superfamily	Total	Max	Total	Max
Immunoglobulin	2752	106	2968	22
EGF/Laminin	2085	19	2142	11
Spectrin repeat	2039	49	1907	48
Fibronectin Type III	1758	29	1779	16
LDL receptor-like module	997	12	1219	23
ARM repeat	858	13	775	12
Beta-beta-alpha zinc fingers	826	23	768	25
Cadherin-like	819	34	857	78
Complement control module/SCR domain	811	37	1025	14
Growth factor receptor domain	696	9	711	7

## 4 Discussion

Here, we present a simulation strategy that mimics the patterns of domain order and adjacency in multidomain architectures over the course of evolution. Our algorithm has been implemented in Python 3 as DomArchov. Domain architecture evolution is governed by a set of constraints that differ fundamentally from those modeled by other simulators used in molecular evolution. As such, our simulator is novel and unique in its conception.

DomArchov is based on a Markov model wherein transition probabilities are estimated using domain bigram frequencies to calculate first-order conditional probabilities. These probabilities depend only on the domain content in the immediate neighborhood of the site of change. Remarkably, although state transitions are based only on local information about domain order and proximity, DomArchov is able to elicit global properties of domain architectures. These include DA length distributions, mean tandem array lengths, the propensity to co-occur at any location in the same domain architecture, not just at adjacent positions, and the ability to reproduce a preference for events at domain architecture termini.

### 4.1 Comparison with other simulation models

Our strategy bears some similarity to the use of word n-gram models in predicting continuations in natural language processing (NLP). Indeed, our pseudocount model is based on smoothing methods used in NLP (Jurafsky and Martin, 2008). However, some NLP techniques based on n-gram statistics are beyond our reach because of the modest size of the domain architecture datasets available for training. For example, domain architecture training data are typically too small to permit evaluation strategies that require separate training and testing datasets. Instead, to offset the risk of circularity, we focus on test statistics selected to be independent from the statistics used to instantiate the model.

Although it may seem that simulation techniques for sequence evolution could be adapted to modeling domain architecture evolution, the entities (biomolecular sequences and domain architectures) are governed by very different sets of mutational processes, selective constraints, and data size. In nucleic and amino acid sequences, substitutions at a single site are the fundamental unit of change. Although more recent models have been expanded to account for indels and site-specific interactions, the core process focuses on replacement of a residue with a different residue at a single site.

In contrast, domain architectures change through domain gain and domain loss, but not domain replacement. In nature, replacement of one sequence segment by another is mediated by homologous recombination, which requires that the sequences be related. This mechanism can, and likely does in some cases, act to replace an instance of a domain superfamily with a different instance of the same superfamily. Such events would indeed change the sequence, but not its DA representation. Domain replacement in the DA model would necessitate the replacement of a sequence segment encoding one domain superfamily with a segment encoding an unrelated superfamily. It is not clear what molecular mechanism would result in a such a replacement in a single event.

### 4.2 Future work

Combining domain architecture simulation with simulation of other aspects of protein evolution is an important next step. There are also important new directions to explore within the restricted context of domain architecture evolution. Currently, a single event is used to model all gains in DomArchov; there is no distinction, e.g. between domain insertion and domain duplication. Expanding DomArchov to distinguish between various mechanisms that result in the addition of a domain is a fruitful area of future research. Modeling the gain or loss of several domains in a single event is another important future direction. This is particularly relevant for realistic simulation of tandem repeat formation, where duplication of several adjacent copies is a frequent occurrence (Björklund *et al.*, 2006, 2010; Han *et al.*, 2007).

From an empirical standpoint, the studies presented here have focused on metazoan lineages. Whether DomArchov’s data-driven functions perform as well in other taxonomic lineages must be examined. This study has focused on establishing convergence and characterizing the steady-state behavior of the simulator. We look forward to probing its behavior on shorter time scales, as well.

## Supplementary Material

btac242_Supplementary_Data
