# Supplementary material for: Simulating domain architecture evolution
Source: Bioinformatics. 2022 Jun 27;38(Suppl 1):i134–42. doi: 10.1093/bioinformatics/btac242 (PMC9236583; doi:10.1093/bioinformatics/btac242)
Supplement: btac242_Supplementary_Data [file btac242_supplementary_data.pdf]

S Supplementary Information

Primates	Fish	Drosophila	Cnidaria
Gorilla gorilla	*Astyanax mexicanus*	*Drosophila ananassae*	*Acropora digitifera*
Mus musculus	*Gasterosteus aculeatus*	*Drosophila melanogaster*	*Hydra vulgaris*
Pongo abelii	*Oryzias latipes*	*Drosophila pseudoobscura*	*Nematostella vectensis*
Homo sapiens	*Tetraodon nigroviridis*	*Drosophila suzukii*	*Thelohanellus kitauei*
Nomascus leucogenys	*Danio rerio*	*Drosophila yakuba*	
Macaca mulatta	*Lepisosteus oculatus*	*Drosophila erecta*	
Pan troglodytes	*Poecilia formosa*	*Drosophila mojavensis*	
	Xiphophorus maculatus	*Drosophila sechellia*	
	Gadus morhua	*Drosophila virilis*	
	Oreochromis niloticus	*Drosophila grimshawi*	
	Takifugu rubripes	*Drosophila persimilis*	
		Drosophila simulans	
		Drosophila willistoni	

Table S1. Genomes used in this study

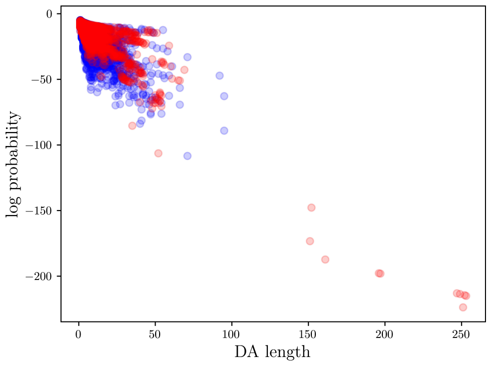

(a) Primates

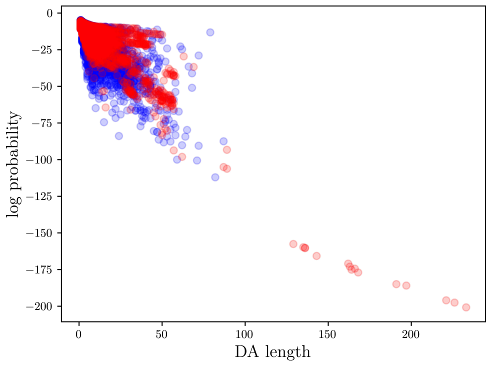

(b) Fish

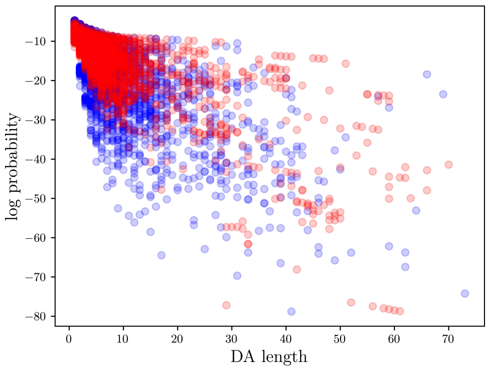

(c) Drosophila

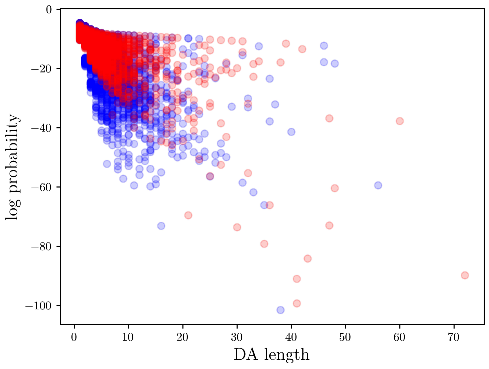

(d) Cnidaria

Fig. S1: Dependence of domain architecture probability on domain architecture length in genuine (red) and simulated (blue, $T = 3.2M$) domain architectures.

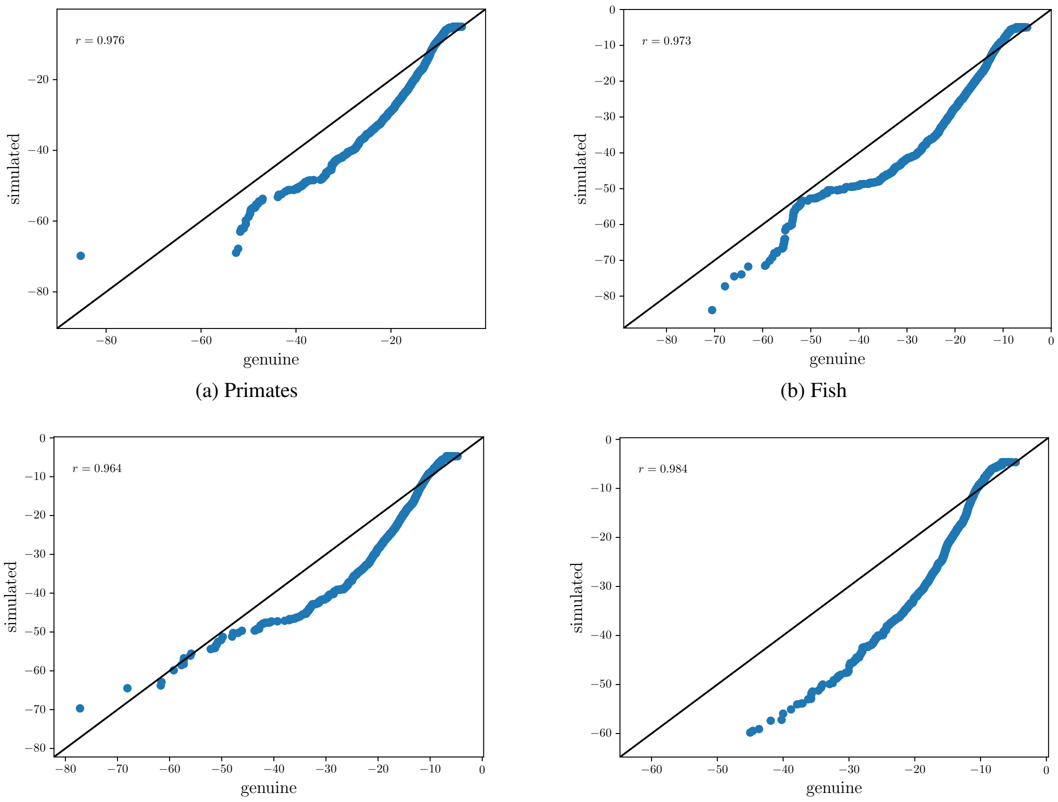

(a) Primates (b) Fish

(c) Drosophila (d) Cnidaria

Fig. S2: Q-Q plot of genuine and simulated ($T = 3.2M$) domain architecture log likelihood, calculated using the first order approximation (Eqn 2) with bigram frequencies in the genuine data. Length outliers (the longest 1% of domain architectures) in each set are not shown.

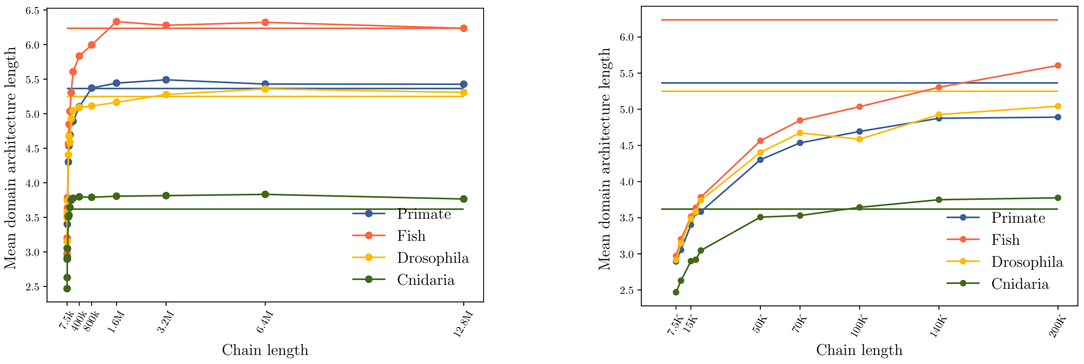

(a) Mean domain architecture lengths (b) Mean domain architecture lengths (detail)

Fig. S3: Final domain architecture length as a function of chain length, averaged over all replicates. Horizontal lines represent mean length of genuine domain architectures.
